# Proactive Aesthetic Strategies: Evaluating the Preventive Role of Botulinum Toxin in Facial Aging

**DOI:** 10.3390/muscles4030031

**Published:** 2025-08-13

**Authors:** Grazia Marinelli, Alessio Danilo Inchingolo, Irma Trilli, Carmela Pezzolla, Roberta Sardano, Francesco Inchingolo, Andrea Palermo, Cinzia Maria Norma Maspero, Gianna Dipalma, Angelo Michele Inchingolo

**Affiliations:** 1 Department of Interdisciplinary Medicine, University of Bari “Aldo Moro”, 70121 Bari, Italy; graziamarinelli@live.it (G.M.); alessiodanilo.inchingolo@uniba.it (A.D.I.); trilliirma@gmail.com (I.T.); carmela.pezzolla@uniba.it (C.P.); roberta.sardano@uniba.it (R.S.); gianna.dipalma@uniba.it (G.D.); angeloinchingolo@gmail.com (A.M.I.); 2 Department of Experimental Medicine, University of Salento, 73100 Lecce, Italy; andrea.palermo@unisalento.it; 3 Department of Biomedical, Surgical and Dental Sciences, Milan University, 20122 Milan, Italy; cinzia.maspero@unimi.it; 4 Unit of Maxillo-Facial Surgery and Dentistry, Fondazione IRCCS Ca’ Grande Ospedale Maggiore Policlinico, 20122 Milan, Italy

**Keywords:** botulinum toxin, preventive aesthetics, skin aging, wrinkle prevention, cosmetic dermatology, neuromodulators, baby botox, facial rejuvenation, intrinsic aging, extrinsic aging

## Abstract

Background: In recent years, botulinum toxin (BoNT) has been increasingly employed not only as a corrective aesthetic intervention but also as a proactive strategy to delay the visible signs of facial aging. This systematic review aims to evaluate the scientific evidence supporting the preventive role of BoNT in facial aging, focusing on its long-term effects, mechanisms of action, and clinical outcomes when used in younger, pre-symptomatic populations. Methods: A systematic literature search was conducted across PubMed, Scopus, and Web of Science databases. Inclusion criteria encompassed clinical trials and observational studies addressing the use of BoNT for proactive aesthetic strategies. Results: Evidence suggests that early BoNT application may reduce muscle hyperactivity, delay the formation of dynamic wrinkles, and minimize the development of static lines over time. Histological studies indicate a potential remodeling effect on dermal collagen. However, data remain heterogeneous, and long-term safety and efficacy outcomes are not yet fully established. Conclusion: Preventive BoNT injections represent a promising tool in the proactive management of facial aging. Further longitudinal, high-quality studies are needed to substantiate its role within evidence-based aesthetic protocols.

## 1. Introduction

### 1.1. Background on Skin Aging

Skin aging is a complex, multifactorial biological process influenced by intrinsic and extrinsic factors [1,2,3,4,5,6]. Intrinsic aging, also known as chronological aging, is a genetically programmed and inevitable process characterized by a gradual decline in physiological functions, including those of the skin [7,8,9,10,11]. Extrinsic aging, on the other hand, is primarily driven by environmental exposures such as ultraviolet (UV) radiation, pollution, smoking, and lifestyle factors, a phenomenon commonly referred to as “photoaging” [12,13,14,15,16,17].

From a histological standpoint, aging skin is marked by several structural changes: epidermal thinning, decreased collagen and elastin production in the dermis, reduction in dermal vasculature, and diminished sebaceous gland activity [18,19,20,21,22,23,24]. These changes manifest clinically as fine lines, wrinkles, laxity, dryness, pigmentation irregularities, and overall loss of skin tone and volume [25,26,27,28,29]. Dynamic wrinkles have traditionally been treated with neuromodulators such as botulinum toxin type A (BoNT-A) [30,31,32,33,34,35,36,37].

### 1.2. Botulinum Toxin: Mechanism and Historical Use

**Figure 1:**
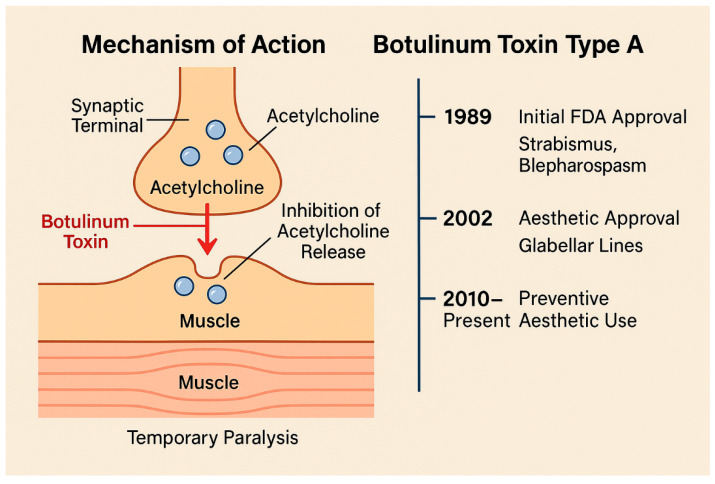
Mechanism of action of botulinum toxin type A at the neuromuscular junction. The toxin blocks the release of acetylcholine, resulting in temporary muscle paralysis. This mechanism underlies its clinical use in aesthetic medicine to reduce dynamic wrinkles caused by facial muscle contractions.

Botulinum toxin is a neurotoxin produced by Clostridium botulinum that temporarily inhibits the release of acetylcholine at the neuromuscular junction, thereby inducing a reversible muscular paralysis (Figure 1) [38,39,40,41,42]. Since its initial FDA approval in 1989 for the treatment of strabismus and blepharospasm. BoNT-A has become an essential tool in aesthetic medicine, particularly following its approval in 2002 for the temporary improvement of glabellar lines [43,44,45,46].

The aesthetic use of BoNT has evolved significantly over the last two decades [47,48,49,50,51,52]. Once considered solely corrective, BoNT is now also used preventively, especially in younger individuals [53,54,55,56,57]. This paradigm shift has sparked growing interest in understanding the efficacy, safety, and long-term implications of BoNT use in younger, often asymptomatic populations [58,59,60,61,62,63].

### 1.3. The Concept of Preventive Aesthetics

Preventive aesthetics refers to the use of medical or minimally invasive interventions to delay or prevent the visible signs of aging before they become established [64,65,66]. The concept draws parallels with preventive medicine, where early interventions aim to halt or slow the progression of disease [67,68,69,70,71,72,73]. This trend is especially popular among Millennials and Gen Z, who prioritize early maintenance [74,75,76,77].

The idea of using BoNT-A preventively is rooted in the understanding that repetitive muscular contractions lead to mechanical stress on the skin and contribute to the breakdown of collagen and elastin fibers, ultimately resulting in static wrinkle formation [78,79,80,81,82]. By reducing the activity of hyperdynamic muscles early in life, BoNT may minimize this biomechanical stress and preserve dermal integrity over time [83].

Furthermore, beyond its well-known aesthetic applications, botulinum toxin (BoNT) has demonstrated significant analgesic properties, particularly in chronic musculoskeletal disorders. This is due to its modulation of pain-related neuropeptides, suggesting shared pathways with its cosmetic action [84].

### 1.4. Emerging Trends and Cultural Shifts

**Figure 2:**
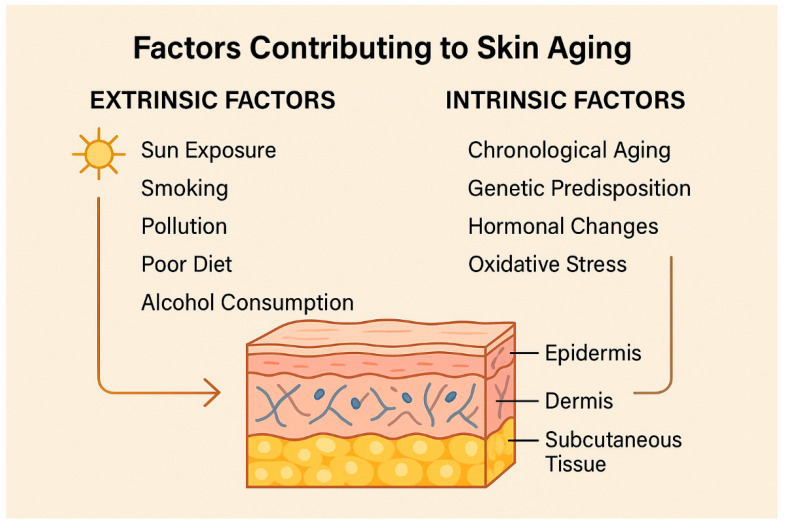
Summary of the extrinsic and intrinsic factors contributing to skin aging.

Social media and celebrity culture have reshaped aesthetic medicine (Figure 2) [85,86,87,88,89,90,91,92]. The visibility of aesthetic procedures and the normalization of cosmetic interventions have led to an increased demand for “prejuvenation”—a portmanteau of “prevention” and “rejuvenation”—wherein individuals seek early treatments not to reverse aging but to prevent it. In this cultural milieu, the preventive use of BoNT has surged, often with patients requesting neuromodulators before any visible lines appear [93,94,95,96].

However, concerns remain about its long-term effects, cost, and psychological impact on young patients. Therefore, rigorous evaluation of the evidence is crucial to guide clinical practice.

## 2. Materials and Methods

### 2.1. Methodology

**Figure 3:**
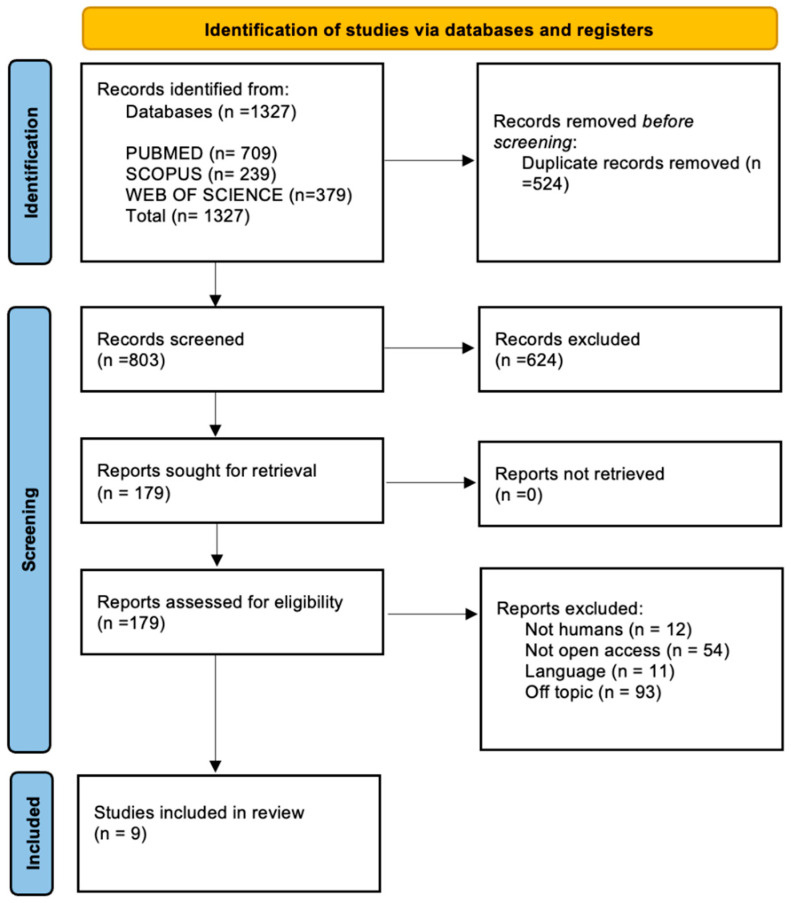
PRISMA flow diagram.

This systematic review was conducted following the PRISMA (Preferred Reporting Items for Systematic Reviews and Meta-Analyses) guidelines to ensure transparency and methodological rigor throughout the research process. The protocol was registered with PROSPERO under the registration number 42025107376. The PRISMA checklist was used to guide each phase of the review, including the development of the search strategy, selection of studies, data extraction, and assessment of methodological quality, thereby minimizing the risk of bias and enhancing reproducibility (Figure 3).

### 2.2. Research Processing

A comprehensive electronic search was conducted in PubMed, Scopus, and Web of Science to identify articles published in English between 2005 and 2025. The search strategy included the following keywords: “botulinum toxin,” “aging,” and “aesthetic treatment”, combined using Boolean operators (AND, OR) to maximize the sensitivity and specificity of the results. For instance, combinations such as “botulinum toxin” AND (“aging” OR “aesthetic treatment”) were used to retrieve articles focusing on both preventive and aesthetic applications of botulinum toxin in facial skin aging.

The search was limited to studies available in English and is open access format due to practical constraints, including institutional access and language proficiency. While this choice ensured full-text availability and transparency, it may also introduce some publication and language bias.

### 2.3. Eligibility Criteria

The selection of studies was based on a PICOS-formulated research question (Table 1):

Population (P): Adult individuals undergoing aesthetic treatment for facial aging.

Intervention (I): Preventive or early application of botulinum toxin (any formulation).

Comparison (C): Untreated control, placebo, or alternative aesthetic interventions.

Outcome (O): Reduction or delay in clinical signs of facial aging, as measured by validated aesthetic scales, objective skin parameters (e.g., hydration, elasticity), or patient-reported outcomes.

Study Design (S): Randomized controlled trials (RCTs), prospective and retrospective cohort studies, and case–control studies.

Based on this PICOS question, the research aim was to assess whether the early aesthetic use of BoNT can effectively prevent or delay the visible signs of facial aging.

The hypothesis was that preventive BoNT treatment in younger or pre-symptomatic individuals leads to improved long-term skin quality and delayed wrinkle formation, compared to untreated or late-treated patients. 

The primary outcome measures included validated wrinkle severity scores, objective skin parameters (e.g., hydration, elasticity), and patient satisfaction or quality-of-life scales.

### 2.4. Exclusion Criteria

The following types of publications and study designs were excluded from this review:

- (1) Animal or in vitro studies, to ensure clinical relevance;
- (2) Review articles, including narrative, systematic, or meta-analyses, to avoid data duplication;
- (3) Studies not evaluating outcomes related to facial rejuvenation or aging prevention;
- (4) Articles not available in English or not accessible in full text.

This approach was aimed at ensuring the inclusion of high-quality primary data directly relevant to the study objective.

### 2.5. Data Extraction and Analysis

Data extraction was carried out independently by two reviewers. Any disagreements were resolved through discussion, and in case of persistent discrepancies, a third reviewer was consulted. Extracted data included study characteristics (authors, year, country), study design, population size and characteristics, botulinum toxin formulation and dosage, treatment protocols, follow-up duration, and outcome measures.

Primary outcome measures included validated aesthetic scales (e.g., Wrinkle Severity Rating Scale), objective skin assessments (e.g., elasticity, hydration), and patient-reported outcomes (e.g., satisfaction scores, quality of life).

The methodological quality of the included studies was evaluated using the Newcastle–Ottawa Scale (NOS) for observational studies and the Cochrane Risk of Bias tool for randomized controlled trials. The results of this assessment were used to gauge the internal validity and risk of bias in the included literature.

A qualitative synthesis of results was conducted. Given the heterogeneity of study designs, treatment protocols, and outcome measures, a meta-analysis was not performed. Instead, results were grouped and compared narratively according to intervention timing (preventive vs. corrective), age group, and outcome type. Patterns, strengths, and gaps in the literature were highlighted to support clinical interpretation and future research directions.

## 3. Results

The electronic search of the three databases identified a total of 1327 studies. Specifically, 709 on PubMed, 379 on Web of Science, and 239 on Scopus. A total of 524 duplicates were identified and removed. After deduplication, all titles and abstracts were screened on 803 articles. Of these, 624 were excluded after checking the relevance of the topic by title and abstract. Finally, 170 studies were excluded for the following reasons:

- Not involving humans (*n* = 12);
- Not available in open access (*n* = 54);
- Language not in English (*n* = 11);
- Off-topic (*n* = 93), meaning not related to the use of botulinum toxin in the context of skin aging or aesthetic facial treatment.

Finally, 9 articles were selected. The selection process is summarized in Figure 3, and the articles enrolled for the Section 4 are summarized in Table 2.

### Quality Assessment and Risk of Bias

The nine studies included in this analysis show variability in study design, sample size, and follow-up duration (Table 3). Using the RoB 2.0 tool (for randomized controlled trials) and a descriptive approach for observational designs, we evaluated the following domains of bias: randomization process, deviations from intended interventions, missing outcome data, outcome measurement, and selection of reported results. Most of the randomized controlled trials (e.g., Zhu, Kim, Dayan, Fabi, Harii, and Kawashima) demonstrate low risk of bias, with proper randomization, blinding procedures, and well-documented outcomes. In contrast, Kapoor et al. [98]. presented a high risk of bias due to the small sample size and lack of statistically significant results. Keen et al.’s results, being an earlier clinical study without a control group, show moderate risk, particularly due to subjective outcome measures and absence of blinding. Overall, the included studies demonstrate high methodological quality, especially among recent phase 3 randomized controlled trials (e.g., Fabi, Harii, Kawashima, and Keaney), which employed strict randomization, blinding, and validated outcome assessments. Kapoor et al. [98]. had significant limitations, primarily due to its small sample size and lack of significant findings, leading to a high risk of bias. Keen et al. (1994) [82] presented moderate bias concerns due to a lack of randomization and reliance on patient-reported outcomes. No critical issues were found regarding selective outcome reporting across all studies. The consistent methodology in most trials enhances confidence in the overall conclusions, though future studies with longer follow-up and larger cohorts are needed to confirm these early findings, especially in intradermal applications of botulinum toxin.

## 4. Discussion

### 4.1. Skin Effects of Intradermal Botulinum Toxin: Hydration, Elasticity and Rejuvenation

Three recent studies (Zhu et al. [97], Kim et al. [35], and Kapoor et al. [98]) have focused on the efficacy of intradermal injection of botulinum toxin A (BoNTA) to improve skin parameters such as hydration, elasticity, TEWL, and general skin quality, going beyond the classical neuromuscular action.

Zhu et al. (1) evaluated the efficacy of intradermal botulinum toxin type A (BoNTA) injections for facial rejuvenation in 40 Chinese women. Participants were randomly assigned to a BoNTA group or a saline control group. Assessments were conducted at baseline, 4 weeks, and 12 weeks post-treatment. At 12 weeks, the BoNTA group showed significantly higher physician-assessed global improvement scores (2.30 ± 0.40 vs. 0.70 ± 0.60, *p* < 0.05) and patient satisfaction scores (2.40 ± 0.50 vs. 0.75 ± 0.47, *p* < 0.05) compared to controls. Biophysical measurements demonstrated that BoNTA significantly improved skin hydration, elasticity, and roughness and reduced transepidermal water loss (TEWL) at 12 weeks, while erythema and melanin indices remained unchanged. The study suggests that intradermal BoNTA promotes facial rejuvenation, likely through dermal collagen synthesis rather than muscle relaxation, with effects emerging over 2–3 months. Despite the promising results, the authors highlight the need for larger, long-term randomized controlled trials to confirm these findings and to compare intradermal versus conventional intramuscular BoNTA administration [97]. Kim et al. (2) evaluated the efficacy and safety of intradermal botulinum toxin (BTX) injections in rosacea patients with facial erythema. Twenty-four participants received intradermal BTX on one cheek and saline on the contralateral cheek. Assessments were performed at baseline and at 2, 4, 8, and 12 weeks, including the Clinician Erythema Assessment (CEA), Global Aesthetic Improvement Scale (GAIS), skin hydration, transepidermal water loss (TEWL), melanin content, erythema index, elasticity, and sebum secretion. The BTX-treated side showed a significant reduction in CEA scores and erythema index (notably at weeks 4 and 8) and a significant increase in GAIS scores. Improvements were also observed in skin elasticity at weeks 2 and 4 and skin hydration at weeks 2, 4, and 8. No significant differences were noted in TEWL or sebum secretion. These results suggest that intradermal BTX is effective and well tolerated in reducing facial erythema and improving skin quality in rosacea patients, supporting its potential as an adjunctive treatment modality [35].

In contrast, Kapoor et al. (3) found no significant differences between the BoNTA-treated side and the saline-treated side, although they showed a subjective improvement in skin texture. Ten physician participants provided informed consent and were treated with intradermal injections of onabotulinumtoxinA (2 U/0.1 mL, 30 injections per half-face) on one side of the face and normal saline on the contralateral side. Both the injecting clinician and participants were blinded to the treatment allocation. Assessments were conducted at 1 and 4 weeks post-treatment by two neutral, blinded observers, who evaluated participants in person and via standardized photographs using a −4 to +4 rating scale. While post-treatment photographs showed global improvements in skin texture and tightness (with skin appearing smoother and more taut), no significant differences were observed between the botulinum toxin and saline-treated sides. Thus, the observed changes could not be attributed specifically to the botulinum toxin injections. No other meaningful clinical differences were detected, and the small sample size limited formal statistical analysis. The findings suggest that intradermal onabotulinumtoxinA injections do not provide a significant benefit for facial rejuvenation. However, the small sample size and lack of statistical significance reduce their strength [98].

These three studies, while sharing the intradermal approach, show variable results, probably related to the number of participants, duration of follow-up, and individual variability of skin response.

### 4.2. Botulinum Toxin for the Treatment of Dynamic and Static Wrinkles

The most established clinical data come from the oldest studies, which mainly investigated the effect of BoNTA on periorbital and glabellar wrinkles through targeted injections.

Keen et al. (4) demonstrated the efficacy of BoNTA in reducing ‘crow’s feet’ wrinkles through electromyography-guided injections. The effect was visible within 72 h and lasted up to 6 months, with significant improvements in 95% of patients. Interestingly, the results were more pronounced in younger subjects (30–50 years), confirming the greater skin plasticity [82].

Harii et al. (6) and Kawashima et al. (7), in recent phase 3 studies (2020–2022), confirmed and strengthened these data with large Japanese samples and repeated treatments over time. Efficacy was constant with dosages of both 12 U and 24 U for periocular wrinkles and 32 U to 44 U for glabellar and periocular combinations. The duration of the effect was maintained at 3–4 months, with minimal side effects and high patient satisfaction.

The time comparison suggests that although administration techniques have been refined over time, the clinical results are consistent between studies from the 1990s and recent studies, showing excellent reproducibility of BoNTA’s efficacy in the treatment of dynamic wrinkles [101,103].

### 4.3. Psychosocial Benefits and Quality of Life

Interest in the emotional and psychosocial effects of the aesthetic use of botulinum toxin has emerged in more recent and structured studies, such as that of Dayan et al. (5), which included validated scales to assess quality of life and self-esteem.

The results indicate a significant improvement not only in physical appearance but also in emotional well-being, body satisfaction, mood, and age perception. Interestingly, the placebo group also showed benefits, underlining the importance of the aesthetic experience as a whole [99].

The study on male patients conducted by Keaney (8) et al. (on more than 1,100 subjects, including 140 men) also confirmed high satisfaction rates and psychological improvements. The effectiveness of the treatment was significant despite the greater muscle mass of the male subjects, with lasting effects for up to 6 months.

These studies suggest that the impact of botulinum toxin goes beyond the aesthetic sphere, involving relational, psychological, and identity aspects, making it a true wellness therapy [102].

### 4.4. Unconventional Treatments: Cervical and Platysmal Region

Finally, a more recent and less explored area concerns the treatment of the cervical region, in particular, the prominence of the platysma muscle.

The study by Fabi et al. (9) (phase 3, multicenter) showed that BoNTA can significantly improve the appearance of the neck, with visible and subjective improvements already at 14 days. Again, the toxin showed a good safety profile, with no dysphonia or dysphagia, typical side effects feared in this area.

This opens the way to new non-facial applications, confirming the versatility of the toxin in the cosmetic field [100].

### 4.5. Future Perspectives on the Use of Botulinum Toxin (BoNT-A)

#### 4.5.1. Personalized and Evidence-Based Approaches

The preventive use of BoNT-A underscores the need for personalized treatment protocols, tailored to individual factors such as muscle activity, skin type, phototype, and genetic predisposition to early wrinkle formation. Future research should focus on developing evidence-based guidelines to support individualized dosing and timing, avoiding a “one-size-fits-all” approach.

#### 4.5.2. Predictive Biomarkers and Treatment Response

Identifying clinical or molecular biomarkers that can predict treatment response, duration of effect, and risk of adverse events would greatly enhance patient selection and treatment planning, particularly in younger and asymptomatic populations.

#### 4.5.3. Prevention with Caution

While “prejuvenation” holds promise, its long-term safety profile remains underexplored. Longitudinal studies are needed to determine whether early intervention may lead to progressive tolerance, neuromuscular adaptation, or any long-term structural changes.

#### 4.5.4. Aesthetic and Therapeutic Synergies

The analgesic properties of BoNT-A suggest potential synergy between aesthetic and therapeutic applications, such as in patients suffering from tension-type headaches, bruxism, or myofascial syndromes, who also exhibit early signs of aging. A multidisciplinary approach could expand treatment indications and improve overall quality of life.

#### 4.5.5. Sustainability and Ethical Considerations in Preventive Aesthetics

The growing popularity of BoNT-A among younger demographics raises questions about cost-effectiveness and ethical practice. Treating wrinkle-free individuals influenced by social pressures or unrealistic beauty standards requires careful patient education and responsible medical judgment to promote realistic expectations.

#### 4.5.6. Emerging Technologies and Alternative Delivery Systems

Future innovations may include controlled-release formulations or non-injectable delivery systems that improve the patient experience and reduce the risks of repeated injections. Additionally, 3D imaging, AI-assisted diagnostics, and dynamic facial analysis could optimize injection site selection and treatment planning.

#### 4.5.7. Expanding Aesthetic Indications

Beyond dynamic wrinkles, BoNT-A is being explored for off-label uses such as reducing enlarged pores, sebum production, facial flushing, and improving skin texture. These emerging applications warrant further clinical investigation and may represent the next frontier in minimally invasive aesthetic dermatology.

## 5. Conclusions

In conclusion, this systematic review highlights the growing interest and promising evidence supporting the preventive use of botulinum toxin in facial aging management. While traditionally employed to reduce established dynamic wrinkles, emerging studies suggest that earlier intervention may delay the onset of visible aging signs by modulating muscular activity, preserving skin elasticity, and improving patient satisfaction. Nevertheless, current evidence remains limited by heterogeneity in study designs, patient selection, outcome measures, and treatment protocols. High-quality longitudinal studies with standardized methodologies are essential to better define the optimal timing, dosage, and long-term efficacy of preventive botulinum toxin treatments. Until such data become available, clinicians should individualize treatment plans based on patient age, skin condition, and aesthetic goals, ensuring a balance between preventive benefits and natural facial expression. From a clinical perspective, the incorporation of preventive botulinum toxin into aesthetic protocols may offer a valuable tool for early anti-aging strategies, while future research should aim to clarify its biological mechanisms and cost-effectiveness in routine practice.

## Figures and Tables

**Table 1:** PICOS framework used for study inclusion.

Criterion	Description
Population	Adults undergoing aesthetic treatment for facial aging
Intervention	Preventive or early application of botulinum toxin
Comparison	No treatment, placebo, or alternative aesthetic interventions
Outcome	Improvement or prevention of facial aging signs (e.g., wrinkles, elasticity)
Study Design	RCTs, prospective/retrospective cohort, case–control, or longitudinal studies

**Table 2:** Enrolled studies in the Section 4.

Authors and Year	Study Type	Materials and Methods	Results
Zhu et al., 2017 [97]	Randomized clinical trial	40 female subjects (33–67 y, Fitzpatrick III-IV) randomized to BoNTA (30 U intradermal) vs. saline control; assessments at baseline, 4, and 12 weeks; subjective, clinical, and biophysical outcomes measured.	At 12 weeks, the BoNTA group showed significantly higher clinical and satisfaction scores; improved hydration, elasticity, roughness, and reduced TEWL; no significant change in erythema or melanin index.
Kim et al., 2019 [35]	Randomized, double-blind, split-face pilot study	24 rosacea patients with facial erythema; intradermal BTX (dose not specified) on one cheek, saline on the other; assessments at baseline, 2, 4, 8, 12 weeks; measured CEA, GAIS, hydration, TEWL, melanin, erythema, elasticity, sebum.	BTX-treated side showed significant reduction in CEA score and erythema index (weeks 4, 8); increased GAIS; improved elasticity (weeks 2, 4) and hydration (weeks 2, 4, 8); no significant changes in TEWL or sebum.
Kapoor et al., 2010 [98]	Interventional, comparative, split-face clinical trial	10 physicians; one half of the face injected with onabotulinumtoxinA 2 U per injection (30 injections total; total dose 60 U), the other half with saline; blinded participants and injectors; assessments at 1 and 4 weeks by blinded observers using in-person evaluation and photographs (−4 to +4 scale).	Post-treatment photos showed global improvement in skin texture and tightness, but no differences between botulinum toxin and saline-treated sides; no meaningful clinical differences; small sample size limited statistical analysis.
Keen et al., 1994 [82]	Clinical study (80 patients)	80 patients (64 F, 16 M; age 21–78); EMG-guided botulinum toxin A injections (5–15 IU per injection, 3 injections per side); standardized photos at baseline, 2, and 12 weeks; patient self-rating (0–3 scale); average follow-up 4–6 months.	95% of patients showed improvement (~34% reduction in wrinkles, 1.36 points on scale); effects visible in 72 h, lasting ~4–6 months; mild side effects (5% transient lower lid droop, 4% bruising); no long-term adverse effects or loss of facial expression reported.
Dayan et al., 2014 [99]	Randomized, Double-Blind, Placebo-Controlled Trial	100 participants (50 BoNTA, 50 placebo) treated for glabellar, frontal, and periocular wrinkles. Treatment efficacy assessed through Q-LES-Q-SF (Quality of Life) and HPSS (Self-Esteem) scales. Follow-up at 2 weeks and 3 months.	BoNTA significantly improved physical appearance, quality of life, and self-esteem compared to placebo. Improvements sustained over 3 months. Participants with no prior treatment showed the greatest benefits.
Fabi et al., 2025 [100]	Phase 3, multicenter, randomized, double-blind, placebo-controlled trial	408 adults with moderate to severe PP were randomized 1:1 to receive onabotulinumtoxinA 26 U, 31 U, or 36 U intramuscularly or placebo. Injections were administered intramuscularly in the platysma. Participants were assessed at Days 14, 30, 60, 90, and 120 for efficacy and safety outcomes.	32.3% of onabotA-treated participants achieved ≥2-grade improvement in PP by Day 14 vs. 1.9% with placebo. A total of 56.9% (clinician) and 74.8% (participant) achieved Grade 1 or 2 severity. Treatment satisfaction (65.9% vs. 11.1%) and psychosocial impact (mean change: −7.4 vs. −1.7) were significantly improved. Adverse events were mild and comparable between groups.
Harii et al., 2017 [101]	Phase 3, multicenter, double-blind, randomized, placebo-controlled, open-label extension	300 Japanese adults; onabotulinumtoxinA 24 U, 12 U, or placebo; 13 months; up to 5 treatments; assessments by Facial Wrinkle Scale with Asian Guide, subject-reported outcomes	68.3% (24 U) and 56.6% (12 U) responders at day 30 vs. 8.2% placebo; duration ~3–4 months; median onset 2–3 days; high subject satisfaction; safe and well-tolerated, no new risks
Keaney et al., 2019 [102]	Post hoc analysis of 2 Phase 3 RCTs	140 male subjects (from 1,178 total) with moderate to severe FHL received 40–64 U onabotulinumtoxinA or placebo. Double-blind for 6 months. Efficacy, PROs (FLSQ, FLO-11), and safety were assessed through Day 180.	98.2% showed ≥1-grade FHL improvement at elevation; 93.3% at rest (Day 30). A total of 81.8% reached “none” or “mild” rating. High satisfaction and psychological improvement. No new safety signals detected.
Kawashima et al., 2019 [103]	Phase 3, multicenter, randomized, double-blind trial	101 Japanese adults (age 20–64); onabotulinumtoxinA 44 U (24 U CFL + 20 U GL) vs. 32 U (12 U CFL + 20 U GL) up to 4 treatments over 13 months; assessed via FWS-A, PROs, and safety metrics	Day 30 CFL responders: 89.6% (44 U), 84.9% (32 U); GL responders: 93.8% (44 U), 98.1% (32 U); median duration: ~114 days; high patient satisfaction; TEAEs mostly mild/moderate; no neutralizing antibodies detected

**Table 3:** Summary of risk of bias in included studies.

Study	Randomization Process	Blinding	Missing Outcome Data	Outcome Measurement	Overall Bias Risk
Zhu et al., 2017 [97]	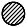	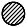	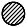	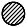	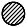
Kim et al., 2019 [35]	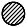	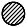	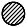	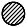	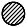
Kapoor et al., 2010 [98]	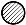	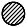	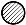	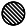	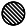
Keen et al., 1994 [82]	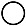	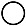	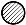	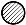	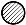
Dayan et al., 2014 [99]	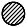	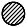	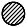	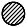	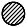
Fabi et al., 2025 [100]	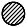	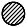	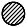	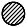	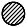
Harii et al., 2017 [101]	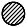	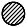	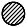	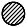	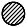
Keaney et al., 2019 [102]	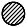	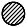	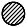	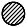	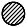
Kawashima et al., 2019 [103]	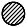	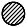	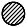	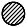	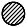

Legend: 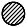 = low risk of bias; 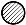 = moderate risk of bias; 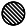 = high risk of bias; 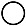 = not applicable/insufficient data.

## Data Availability

No new data were created or analyzed in this study.

## Abbreviations

The following abbreviations are used in this manuscript:

ANLFQ	Appearance of Neck and Lower Face Questionnaire
BAS-PP	Bother Assessment Scale—Platysma Prominence
BoNTA	Botulinum Toxin Type A
BTX	Intradermal Botulinum Toxin
C-APPS	Clinician Allergan Platysma Prominence Scale
CEA	Clinician Erythema Assessment
CFL	Crow’s Feet Lines
CI	Confidence Interval
FHL	Forehead Lines
FLO-11	Facial Line Outcomes Questionnaire
FLSQ	Facial Line Satisfaction Questionnaire
FWS-A	Facial Wrinkle Scale with Asian Photonumeric Guide
GAIS	Global Aesthetic Improvement Scale
GL	Glabellar Lines
HPSS	Health-Related Quality of Life Psychological Self-Perception Scale
ITT	Intent-To-Treat
IU	International Units
Q-LES-Q-SF	Quality of Life Enjoyment and Satisfaction Questionnaire—Short Form
mITT	Modified Intent-To-Treat
onabotA	OnabotulinumtoxinA
P-APPS	Participant Allergan Platysma Prominence Scale
PP	Platysma Prominence
PROs	Patient-Reported Outcome
RCT	Randomized Controlled Trial
TEAE	Treatment-Emergent Adverse Event
TEWL	Transepidermal Water Loss
U	Units (of botulinum toxin and for dosage of BoNTA)
